# Comparative genome analysis of 15 clinical *Shigella* *flexneri* strains regarding virulence and antibiotic resistance

**DOI:** 10.3934/microbiol.2019.3.205

**Published:** 2019-08-13

**Authors:** Liang Wang, Zuobin Zhu, Huimin Qian, Ying Li, Ying Chen, Ping Ma, Bing Gu

**Affiliations:** 1 Department of Bioinformatics, School of Medical Informatics and Engineering, Xuzhou Medical University, Xuzhou 221000, Jiangsu China; 2 Jiangsu Key Laboratory of New Drug Research and Clinical Pharmacy, Xuzhou Medical University, Xuzhou 221000, Jiangsu China; 3 Department of Genetics, School of Life Sciences, Xuzhou Medical University, Xuzhou, Jiangsu, China; 4 Jiangsu Provincial Center for Disease Control and Prevention, Nanjing 210009, China; 5 Medical Technology School of Xuzhou Medical University, Xuzhou 221004, China; 6 Department of Laboratory Medicine, Affiliated Hospital of Xuzhou Medical University, Xuzhou 221006, China

**Keywords:** *Shigella*, virulence factor, comparative genomics, antibiotics resistance, HMMER, Prokka

## Abstract

Shigellosis is the major cause of dysentery globally. It is mainly attributed to two *Shigella* species, *Shigella sonnei* and *Shigella flexneri*, which leads to approximately 165 million infections and 1.1 million deaths each year. Rapid increase and widening of spectrum in antibiotics resistance make *Shigella* hard to be adequately controlled through existing prevention and treatment measures. It has also been observed that enhanced virulence and advent of antibiotic resistance (AR) could arise almost simultaneously. However, genetic linkages between the two factors are missing or largely ignored, which hinders experimental verification of the relationship. In this study, we sequenced 15 clinically isolated *S. flexneri* strains. Genome assembly, annotation and comparison were performed through routine pipelines. Differential resistant profiles of all 15 *S. flexneri* strains to nine antibiotics were experimentally verified. Virulence factors (VFs) belonging to 4 categories and 31 functional groups from the Virulence Factor Database (VFDB) were used to screen all *Shigella* translated CDSs. Distribution patterns of virulence factors were analysed by correlating with the profiles of bacterial antibiotics resistance. In addition, multi-resistant *S. flexneri* strains were compared with antibiotic-sensitive strains by focusing on the abundance or scarcity of specific groups of VFs. By doing these, a clear view of the relationships between virulence factors and antibiotics resistance in *Shigella* could be achieved, which not only provides a set of genetic evidence to support the interactions between VFs and AR but could also be used as a guidance for further verification of the relationships through manipulating specific groups of virulence factors.

## 1. Introduction

Shigellosis is an acute gastroenteritis infection that leads to approximately 165 million infections and 1.1 million deaths per annum [1]. Most of the deaths are related to children in the age group of less than 5 years old [2]. Shigellosis is caused by facultatively anaerobic, non-motile Gram-negative, rod-shaped bacteria belonging to the genus *Shigella*. *Shigella* infection involves invasion and replication within the colonic epithelium, resulting in severe inflammation and epithelial destruction [3]. There are four groups of *Shigella* in the genus, which includes *Shigella dysenteriae*, *S. boydii*, *S. flexneri* and *S. sonnei*. Each group is further classified into serotypes and sub-serotypes based on their lipopolysaccharide O-antigen repeats [3]. Epidemiological studies showed that *S. dysenteriae* was a dominant cause of large epidemics in the past and is now rarely found while *S. boydii* is infrequently isolated [4]. On the other hand, *S. flexneri* and *S. sonnei* are the major causes for Shigellosis. However, the two *Shigella* strains show distinct geographic distribution patterns, which relies on the socioeconomic conditions of the area. Specifically, *S. sonnei* is more tightly linked to countries with higher human development index such as Europe and North America while *S. flexneri* dominates in the low-income regions such as Africa and some of the Asian countries [3]. With the improvement of socioeconomic conditions, transition of dominant strains from *S. flexneri* to *S. sonnei* was also observed [5].

*S. flexneri* and *S. sonnei* were earlier susceptible to a spectrum of antibiotics. New drug resistant phenotype normally develops within a decade of their release [3]. However, due to antibiotics abuse, drug or multi-drug resistant (MDR) strains emerge more frequently than ever [6]. In addition, international travellers and unprotected sex between men increase the dissemination of *Shigella* across countries and lead to potential increase of antibiotic resistance [3]. Historically, *Shigella* was treated with antibiotic drugs such as sulphonamides, tetracycline, and chloramphenicol successively [7]. Antibiotics such as ampicillin, co-trimoxazole, nalidixic acid, and fluoroquinolones were then introduced for combating the bug due to resistance to former drugs [7]. When fluoroquinolones resistant *Shigella* strains emerged, stronger drugs like ceftriaxone, pivmecillinam, and azithromycin were used for treating the infection [7]. Thus, MDR *Shigella* strains present a heavy burden and emerging threats to the society. Common strategies of bacterial antibiotic resistance include but not limited to reduced drug penetration, antibiotics efflux, target modification by mutation and antibiotics hydrolysis [7]. Currently, many shigellosis outbreaks are linked to resistant *Shigella* strains [7]. Rapid increase and widening of spectrum in antibiotics resistance makes *Shigella* hard to be adequately controlled by existing prevention and treatment measures [8]. Thus, endeavours have been tried to accelerate the development of *Shigella* vaccines. Vaccine antigens, *Shigella* subunit vaccines, live oral *Shigella* vaccines, and also killed whole-cell oral *Shigella* vaccines are currently under development [3].

*Shigella* spp. were evolved from non-pathogenic *E. coli* ancestors by acquisition of chromosomal pathogenicity islands and a large virulence plasmid while genes related with anti-virulence such as *cadA* and *ompT*, bacterial mobility like flagella and fimbriae, and catabolism were lost [9]. Pathogenicity of *Shigella* virulence mainly involves Type III secretion system (T3SS), adherence, invasion, intracellular mobility and spread, immune system manipulation and evasion, and toxin, *etc* [10]. Accepted paradigm indicated that increased antibiotic resistance is associated with fitness costs, resulting in reductions in *in vivo* virulence [11]. However, experimental validation of this accepted paradigm is modest. Recent studies suggested that there may be a complex interplay between bacterial virulence and resistance [12]. It has been observed that enhanced virulence and advent of antibiotic resistance often arise almost simultaneously [12]. In addition, a global sensory-transduction system BfmRS in *Acinetobacter baumannii* controls both enhanced virulence and resistance [13]. Accordingly, loss of aminoglycoside resistance regulator, AmgRS, was found to enhance aminoglycoside action against bacteria while reducing bacterial virulence [14]. Moreover, an experimental study found that increase in antibiotic resistance might be exacerbated by fitness advantages that enhance virulence in drug-resistant microbes, which was consistently verified in three pathogenic bacteria *Pseudomonas aeruginosa*, *Acinetobacter baumannii* and *Vibrio cholerae* [15]. Although recent progresses suggested that resistance and virulence might be coupled, genetic linkages between the two factors are still insufficient and largely ignored, which hinders further experimental verification of the relationship.

In this study, we collected 15 *S. flexneri* strains from 7 municipal Centers for Disease Control and Prevention (CDC) in Jiangsu province. Profiles of resistance to 9 antibiotics, that is, Amoxicillin/Clavulanic acid (AMC), Ceftiophene (CFT), Cefotaxime (CTX), Gentamicin (GEN), Nalidixic acid (NAL), Norfloxacin (NOR), Tetracycline (TBT), and compound Sulfamethoxazole (SMZ), were experimentally verified. All strains were sequenced via next-generation high-throughput sequencing platform, which were then analysed for core-/pan-genomes and phylogenomic relationships. Distribution patterns of virulence factors under 4 categories belonging to 31 functional groups were studied. Differential distribution patterns of virulence factors were observed in sequenced strains by comparing antibiotic sensitive and resistant strains. In addition, abundance of specific groups of virulence factors and extent of resistance were also correlated, which may provide genetic support for the positive relationship between virulence and resistance.

## 2. Methods and materials

### 2.1. Bacterial isolates, growth conditions and DNA extraction

*Shigella* is currently categorized as class B infectious disease in China. Pathogenic bacteria detected in local hospitals should be reported to the provincial CDC by municipal CDC. Through collaboration with provincial CDC, 15 *Shigella* *flexneri* strains from different patients with either diarrhea or dysentery in different hospitals of 7 municipal cities were isolated by using routine biochemical techniques. Resistance profile to 9 antibiotics (AMC, CFT, CTX, GEN, NAL, NOR, TBT and SMZ) as previously described for each strain was provided by CDC based on their routine screening procedures. Isolates of *Shigella* *flexneri* were plated on trypticase soya agar (TSA). Picked-up single colony was then inoculated in 5ml trypticase soya broth (TSB) and incubated overnight at 37 °C with shaking rate of 200 rpm. DNA isolation was performed using Easy-DNA™ Kit for genomic DNA isolation (Invitrogen Life Technologies, Carlsbad, CA, USA)

### 2.2. Genome sequencing, assembly, and annotation

Genomes of the 15 *Shigella* flexneri isolates were sequenced at Beijing Genome Institute (BGI) in Shenzhen, China by using next-generation sequencing platforms. Genomic DNA (1.5 µg) was fragmented in a microTube using M220 Focused Ultrasonicator (Covaris Inc., Woburn, MA, USA), which were then validated by average molecule length using the Agilent 2100 bioanalyzer instrument (Agilent DNA 1000 Reagents) and quantified by real-time quantitative PCR (qPCR). The qualified libraries were amplified within the flow cell on the cBot instrument for cluster generation (Hiseq 4000 PE Cluster Kit Illumina). The clustered flow cell was loaded onto the Hiseq 4000 Sequencer for paired-end sequencing (Hiseq 4000 SBS Kit, Illumina) with recommended read lengths 100bp or 150bp. Obtained sequences were assessed via FastQC, assembled via SPAdes [16], recorded and reordered via MAUVE [17] based on reference genome *S. flexneri* 2a str. 301 by following Edwards and Holt's beginner's guide to comparative bacterial genome analysis using next-generation sequence data (Version 2) [18]. For the annotation process, assembled DNA sequences of the new draft genomes from the 15 isolates were run through an automatic annotation pipeline via Prokka (rapid prokaryotic genome annotation), followed by manual curation in some cases [19]. Ten files were generated in the specified output directory, such as FASTA file of translated coding genes (protein), FASTA file of all genomic features (nucleotide), and Genbank file containing sequences and annotations, *etc*.

### 2.3. Orthologous gene prediction and genome sequence comparison

Core-/pan-genome analysis was performed by using standalone software Roary [20]. Core genes (99% ≤ strains ≤ 100%), soft core genes (95% ≤ strains < 99%), shell genes (15% ≤ strains < 95%) and cloud genes (0% ≤ strains < 15%) were calculated. Core and unique genes in the genomes were illustrated in Venn diagram. *S. flexneri* genomes were visualized in circular form genome by comparing to the reference genome *S. flexneri* 2a str. 301 via standalone software BRIG [21]. Bacterial analysis pipeline from Center for Genomic Epidemiology (https://cge.cbs.dtu.dk/services/cge/index.php) was used to compare the genomes. Only pre-assembled contig files were submitted to the online server. Theoretical distributions of antibiotic resistance genes and virulence genes were identified, together with plasmid sequences. Multilocus sequence type (MLST) was also performed based on seven housekeeping genes *adk*, *fumC*, *gyrB*, *icd*, *mdh*, *purA*, and *recA*.

### 2.4. Phylogenomic analysis and tree visualization

A Newick tree for 15 *Shigella flexneri* strains, together with another 12 *Shigella sonnei* strains (unpublished data), was generated based on 3052 core genes in each genome by the phylogenomic analysis with default 1000-time bootstrapping tests via FastTree incorporated in software Roary [22]. The tree was then visualized through online webserver interactive Tree of Life (iTOL) [23]. Genome size, number of MDR, and antibiotic resistance profiles for each strain were then added to the tree by using multi-bar and binary templates in iTOL server.

### 2.5. Identification and comparison of putative virulence factors in the Shigella genomes

31 groups of bacterial virulence factors that belong to four categories were downloaded from the Virulence Factor Database (VFDB) [24]. These virulence factors were then used to screen *Shigella* translated CDSs via phmmer command (E-value < 0.00001) in HMMER package [25]. For each group of virulence factors, multiple homologous sequences were found in corresponding proteomes. These homologous sequences were then processed to get rid of redundant sequences. MDR (resistance to more than 1 antibiotics) and sensitive *S. flexneri* strains (resistance to 0 antibiotics) were compared in terms of the abundance of specific groups of virulence factors via in-house Python scripts.

### 2.6. Correlational analysis between virulence and antibiotics resistance

The total number of non-redundant putative virulence factors in each group for each proteome was calculated. Distinct distribution patterns of virulence factors were observed. Correlation between virulence and resistance were further studied by principal component analysis (PCA), which clustered sensitive and resistant strains separately based on the differences of virulence factors.

## 3. Results

### 3.1. Collection of clinically isolated Shigella strains

**Figure 1.**
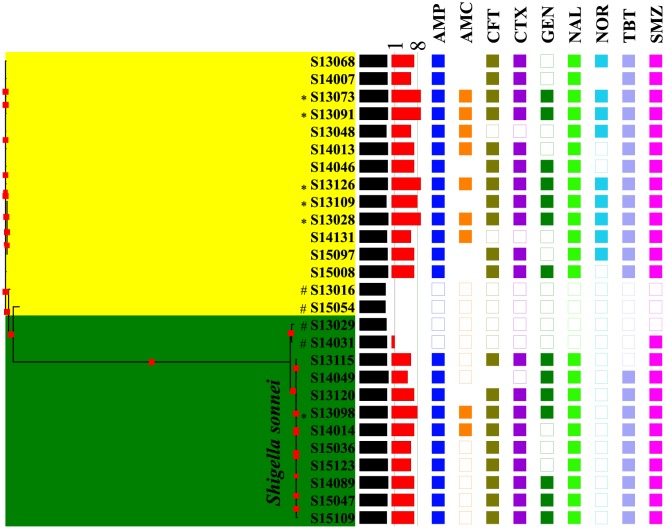
Phylogenomic tree generated via core genes of 27 Shigella strains, which divided *S. sonnei* and *S. flexneri* into two branches. Genome size (black bar) and degree of antibiotic resistance (red bar) were incorporated, accordingly. Bootstrapping values (1000 times) were visualized through red square symbols of varying size on the branches. The presence (filled squares) and absence (empty squares) of nine antibiotics that were tested in this study, which included Amoxicillin/Clavulanic acid (AMC), Ceftiophene (CFT), Cefotaxime (CTX), Gentamicin (GEN), Nalidixic acid (NAL), Norfloxacin (NOR), Tetracycline (TBT), and compound Sulfamethoxazole (SMZ), were presented. No square means intermittent level of resistance. AMC and NOR shows the most apparent resistance difference between *S. flexneri* and *S. sonnei*. ^#^ Most sensitive strains with MDR value of 0 and 1 (vertical line 1). *Most resistant strains with MDR value of 8 and 9 (vertical line 8).

15 *Shigella flexneri* strains with different antibiotic resistance profiles were isolated from 7 cities in Jiangsu province of China. Another 12 strains belonging to *S. sonnei* were also collected and used here only as a comparison for phylogenomic study. Four serotypes of *S. flexneri* were experimentally verified, which includes F1a (4 strains), F1b (1 strain), F2a (8 strains), and F2b (2 strains). Sequence type ST245 was identified in these *Shigella* isolates according to MLST based on seven housekeeping genes. As for antibiotic resistant profiles of the 27 strains, MDR strains ranges from 5 to 9 drug resistance while no or single resistance strains were considered as sensitive. A phylogenomic tree was constructed via core genomes of studies strains, which was incorporated with genome sizes and resistance profiles (Figure 1). Distinct features were observed between the two bacterial strains. A clear genome reduction was identified in sensitive strains when compared with MDR strains (P-value < 0.05). In addition, all *S. sonnei* strains are sensitive to Norfloxacin (NOR) and more labile toward Amoxicillin/Clavulanic acid (AMC) when compared with *S. flexneri*.

### 3.2. Genome assembly, annotation, and comparison

General features of the 15 *S. flexneri* genomes are presented in Table 1, which were obtained by integrating genome assembly and annotation results. Genome size ranges from 4.21 Mbps to 4.63 Mbps. The number of predicted protein-encoding open reading frames (ORFs) in the 15 isolates varied from 4160 (S15054) to 4608 (S13028). The total GC content ranges from 50.38% to 50.78% and is relatively consistent among isolates. All strains have a single tmRNA coding gene. The number of ribosome RNA (rRNA) and transfer RNA (tRNA) coding genes among strains varies slightly with no significant difference.

**Table 1.** Comparison of 15 *S. flexneri* strains based on key genome assembly and annotation parameters.

ID	Serotype	BPs^#^	N50	Contigs	CG%^#^	CDS^#^	tmRNA^#^	tRNA^#^	rRNA^#^
S13016	F2a	4253276	24106	379	50.75	4173	1	80	6
S13028	F2b	4633307	28586	418	50.38	4608	1	79	5
S13048	F1a	4354182	30242	320	50.7	4305	1	81	6
S13068	F2b	4480462	30192	356	50.5	4434	1	81	6
S13073	F1a	4608113	29656	382	50.5	4577	1	79	5
S13091	F2a	4586630	28583	403	50.47	4538	1	81	5
S13109	F2a	4387425	22591	472	50.66	4309	1	78	6
S13126	F2a	4616160	19223	600	50.52	4532	1	81	4
S14007	F2a	4542085	29286	391	50.45	4487	1	77	5
S14013	F2a	4601323	29308	398	50.47	4554	1	80	5
S14046	F1a	4557758	29656	394	50.46	4520	1	81	5
S14131	F2a	4475708	29656	360	50.42	4417	1	79	6
S15008	F1a	4608066	30073	381	50.48	4581	1	80	5
S15054	F1b	4212908	31762	292	50.78	4160	1	79	6
S15097	F2a	4542196	30475	371	50.4	4507	1	79	5

^#^BPs: Base pairs; CDS: coding sequences; GC%: Percentage of GC pairs. tmRNA: transfer-messenger RNA gene. tRNA: transfer RNA gene. rRNA: ribosomal RNA gene.

**Figure 2.**
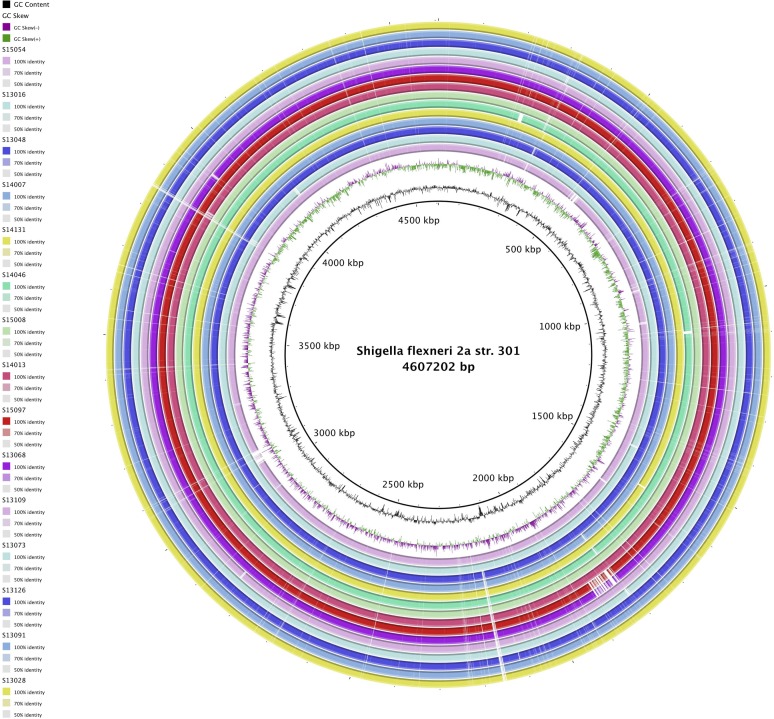
Genome comparison of 15 isolated *S. flexneri* strains against reference genome *S. flexneri* 2a str. 301 generated by BRIG 0.95. The inner cycle (black) represents the complete genome of the reference strain and the shade of each colors denote the similarities between each strain with reference strain. GC content and GC skew (+/-) were illustrated in-between.

By using progressive Mauve from the Mauve software, we compared the ordered genome assembly of *S. flexneri* with *S. flexneri* 2a str. 301 as a reference genome. It seems that the chromosomal alignments of these strains are approximately identical. Additional genomic features of the 15 *S. flexneri* strains against reference genome *S. flexneri* 2a str. 301, such as sequence similarity and distribution of GC content were also analyzed and presented in Figure 2, which indicated that *S. flexneri* genomes are comparatively well reserved among strains.

### 3.3. Core- and pan-genome of 15 Shigella flexneri isolates

**Figure 3.**
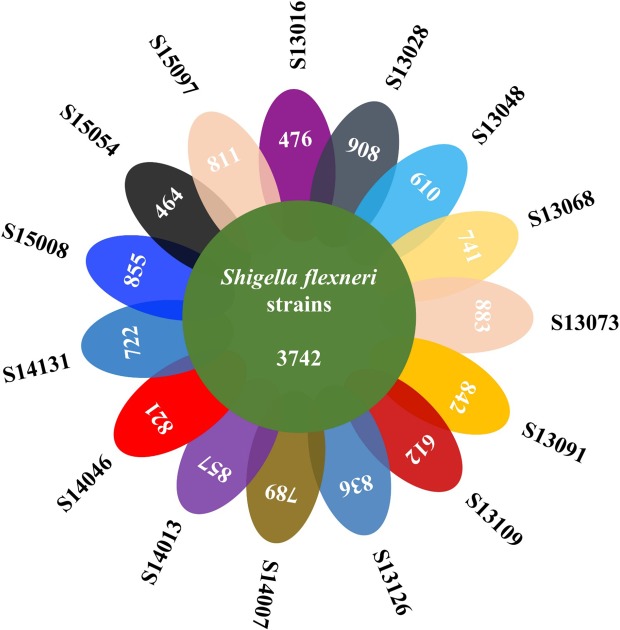
Venn diagram of core- and pan-genome of 15 *S. flexneri* strains. 3742 core genes were shared by all strains while varied number of genes were present in each strain as unique genes. The two sensitive strains (S13106 and S15054) have the lowest number of unique genes (476 and 464) while the two most resistant strains (S13028 and S13073) have the highest number of unique genes (908 and 993).

Core and pan-genome analyses for 15 *S. flexneri* isolates were determined by Roary through comparison of the translated CDS set, followed by clustering of orthologous proteins and the representatives of each orthologous cluster and strain-specific CDS in the total pan-genome. The total pan-genome for the 15 compared *S. flexneri* strains encompasses 5626 CDS. Of these, 3742 (66.51% of total CDS) are core conserved genes across all 15 *Shigella* genomes. A total of 1884 protein CDS (33.49% of the pan-genome total) constitute the accessory fraction, which are unique to each genome. The lowest numbers of specific genes were encoded by *S. flexneri* strains S13016 and S15054, with 476 and 464, respectively. The highest numbers of specific genes belong to *S. flexneri* strains S13028 and S13073, with 908 and 883, respectively (Figure 3). Interestingly, the former two strains are completely sensitive to the nine antibiotics while the latter two strains are resistant to all the tested antibiotics. This is consistent with theoretical prediction of antibiotic resistance genes (ARGs) via Bacterial Analysis Pipeline from Center for Genomic Epidemiology, in which S13016 has no resistance genes and S15054 was found to harbour a single resistance gene *sul2* only. In contrast, other MDR strains have abundant ARGs. In addition, MDR *S. flexneri* strains are commonly equipped with virulence factors such as *capU*, *gad*, *ipaD*, *lpfA*, *pic*, *sepA*, *sigA*, and *virF*. In contrast, sensitive strains only have partial set of these genes, that is, *gad*, *lpfA*, *pic*, and *sigA*. Theoretical analysis found that S13016 has no known plasmid while S15054 harbors plasmid replicons of Col(MG828) and ColRNAI with no known typing. On the other hand, MDR strains have replicon typing IncN, IncI, and IncF except for S13048. For details, please refer to Table S1.

### 3.4. Distribution of virulence factors among S. flexneri strains

**Figure 4.**
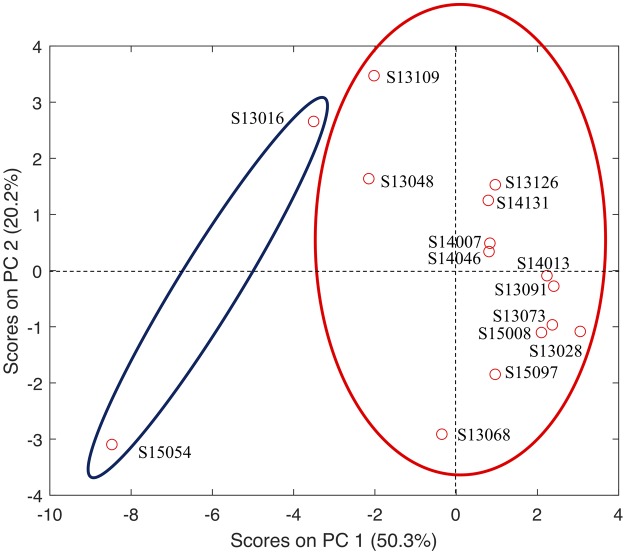
Principal component analysis (PCA) of the relationship between antibiotic resistance and virulence factors in 15 sequenced *Shigella flexneri* strains. S13016 and S15054 are antibiotics-sensitive strains while the rest strains are multi-drug resistant. The two sensitive strains have comparatively less virulence factors in a majority of functional groups while resistant strains have comparatively higher number of certain groups of virulence factors. Thus, resistance and virulence could have a mutual improvement relationship, although exceptions do exist.

*In silico* identification of the putative virulence genes were performed on the translated CDSs of all isolated *S. flexneri* strains. All the putative virulence factors were classified into 4 major categories, that is, adhesion and invasion, secretion system and effectors, toxin, and iron acquisition, which were further divided into 31 functional groups. Distribution patterns of the virulence factors and their abundance in each strain were presented in Table 2. Nine groups of virulence factors are completely missing in all *S. flexneri* strains, which are β-PFTs (pore-forming toxin), superantigens and superantigen like protein, surface acting enzymes, glucosyltransferase, guanylate adenylate cyclase, deamidase, rRNA N-glycosidase, metalloprotease, intracellular PFTs. Five groups of virulence factors, that is, sortase assembled pili, fibrinogen-binding protein, collagen-binding protein, T7SS, and ADP Ribosyltransferase, are highly conserved and equally distributed in these strains. For the rest of the 17 functional groups, most of them were skewedly distributed in highly resistant strains (MDR = 9) when compared with sensitive strains, especially for Chaperone usher and T3SS. In order to better understand the relationship between resistance and virulence, principal component analysis was performed. Although many factors interfere, an apparent cluster could be observed for sensitive and resistant strains in terms of abundance of functional groups of virulence factors (Figure 4).

**Table 2.** Distribution patterns of 4 categories of virulence factors that belong to 31 groups among 15 *Shigella flexneri* strains in terms of antibiotic resistance. Sensitive strains have no resistance or only resist to one antibiotics. The four categories of VFs are Adhesion & Invasion, Secretion system & Effectors, Toxin, Iron acquisition. MDR strains have more than one resistance. PTS: Pore-forming toxins. It is noteworthy that the number of virulence factors represents the number of hits identified in bacterial proteomes via blastp.

	*Shigella flexneri* Strains	S13016	S15054	S13048	S14007	S14131	S13068	S14013	S14046	S15008	S15097	S13109	S13028	S13073	S13091	S13126
	MDR	0	0	6	6	6	7	7	7	7	7	8	9	9	9	9
Adhesion & Invasion	Chaperone usher	129	131	135	137	135	134	136	137	138	137	135	138	138	137	135
	Extracellular nucleation precipitation	14	13	14	14	14	14	14	14	14	14	14	14	14	14	13
	Type 4 pili	122	130	125	129	127	135	142	130	140	135	124	138	140	139	129
	Flagella	192	192	191	197	198	197	198	197	197	196	192	198	197	197	197
	Autotransporter	17	13	17	19	19	19	19	19	19	19	18	19	19	19	19
	Fibronectin-binding protein	51	45	51	53	53	49	53	53	53	50	53	53	53	53	53
	Other adherence invasion related VFs	62	59	60	61	61	57	62	61	61	58	63	61	61	61	60
Secretion system & Effectors	T2SS	8	8	8	9	9	13	12	9	13	13	8	14	13	13	9
	T3SS	159	157	161	214	211	213	214	217	218	210	168	218	217	215	212
	T4SS	32	33	31	33	32	40	41	35	42	41	32	53	41	41	34
	T5SS	18	14	18	20	20	20	20	20	20	20	19	20	20	20	20
	T6SS	152	152	153	154	154	152	154	153	154	154	152	154	154	154	155
Toxin	α-PFTs	2	2	2	2	2	2	2	2	2	2	1	2	2	2	1
	Dnase1 genotoxin	29	27	29	28	29	28	29	28	28	28	29	29	28	29	29
	MDR	0	0	6	6	6	7	7	7	7	7	8	9	9	9	9
Iron acquisition	Siderophore mediated iron uptake	272	259	275	277	277	273	279	277	277	274	275	278	279	279	280
	Heme-mediated iron uptake	109	105	108	108	108	106	108	108	108	108	108	108	108	108	108
	Transferrin and lactoferrin mediated iron uptake	3	2	3	3	3	3	3	3	3	3	2	3	3	3	3

^#^Nine groups of virulence factors are not present in all *S. flexneri* strains, which are β-PFTs, Superantigens and superantigen like protein, Surface acting enzymes, Glucosyltransferase, Guanylate adenylate cyclase, Deamidase, rRNA N-glycosidase, Metalloprotease, and Intracellular PFTs. *Five groups of virulence factors have equal number of virulence factors in all strains, which are Sortase assembled pili (n = 1), Fibrinogen-binding protein (n = 3), Collagen-binding protein (n = 8), T7SS (n = 1) and ADP Ribosyltransferase (n = 1).

### 3.5. Genes exclusively associated with antibiotic resistant and sensitive strains

The gene presence and absence matrix for all *S. flexneri* strains were produced by Roary. A complete list of total genes for all strains were listed against 15 S. flexneri strains with 0 as absence and 1 as presence (Table S2). Function for translated protein is also annotated except for hypothetical proteins. By using filter function in Excel table, genes that are exclusively associated with resistant strains (12 genes) or sensitive strains (9 genes) were selected and presented in Table 3.

**Table 3.** Genes exclusively associated with antibiotic resistant and sensitive strains, respectively. Corresponding functions were obtained from UniProt database. Each gene was assigned into functional groups of virulence factors if there is any match.

*S. flexneri*	Gene	Functions	VF Groups
Resistant strains	*aidB_2*	Putative acyl-CoA dehydrogenase	DNase I genotoxin
	*bisC*	Biotin sulfoxide reductase	-
	*dhfrI*	Trimethoprim resistance protein	-
	*flgE*	Flagellar hook protein	Flagella
	*tnsA*	Transposon Tn7 transposition protein	-
	*tnsB*		
	*tnsC*		
	*tnsE*		
	*wecD_2*	dTDP-fucosamine acetyltransferase	-
	*xerC_4*	Tyrosine recombinase	Chaperone usher pathway
	*xerD_3*		
	*ydiN_1*	Amino acid/amine transport protein	-
Sensitive Strains	*alkA*	DNA-3-methyladenine glycosylase	-
	*bcsC_1*	Cellulose synthase subunit	-
	*dgcE_1*	Putative diguanylate cyclase	Type IV pill
	*dgcE_2*		
	*dgcE_3*		
	*dnaK_2*	Putative chaperone	Adherence and invasion
	*dnaK_3*		
	*pgrR_3*	HTH-type transcriptional regulator	Siderophore mediated iron uptake
	*ycaM*	Inner membrane transporter	-

^#^Genes with unknown functions are not included.

## 4. Discussion

15 newly isolated and completely sequenced *S. flexneri* strains were thoroughly analysed in terms of distributions of virulence factors. Although classical thoughts support that virulence and resistance are negatively related, more evidence suggested that virulence and resistance could be enhanced simultaneously [11],[12],[15]. Initial phylogenomic analysis separated sensitive and resistant strains into different clusters (Figure 1), which reflected the intrinsic differences of evolutionary pathways between the two groups. Genome sizes of sensitive and resistant groups also show apparent difference, that is, smaller genomes (4167 CDSs on average) associated with sensitive strains and larger genome (4490 CDSs on average) linked to resistant strains. Similarly, another study focusing on sensitive and resistant *E. coli* isolates also found that more antibiotic sensitive Sudanese strain have smaller genome size while the genome of the resistant Chinese strain is larger [26]. Physically, it is rather difficult for bacteria to develop genetic systems with small genomes. In fact, it has been observed that multidrug resistance phenotype is a function of genome size based on comparative analysis of 22 bacterial species, which is also known as the ‘size matters’ hypothesis [27].

Core-/pan-genome analysis identified that *S. flexneri* strains have different number of unique genes except for the 3742 shared core genes, which reflects the heterogeneity within the same strains. In addition, the two sensitive strains, S13106 and S15054, have the lowest number of unique genes (476 and 464) while bacteria with the highest number of unique genes (908 and 993) are two most resistant strains, S13028 and S13073 (Figure 3). Specific genes in these strains could reflect bacterial characteristics and dynamics, leading to a better understanding of epidemiological features of *S. flexneri* [28]. Insights into these genes are out of the scope of this study and will be explored for future studies.

As for the distribution patterns of functional groups of virulence factors in *S. flexneri* strains, specific patterns were observed, which may provide evidence to support the positive correlation between increased virulence and enhanced antibiotic resistance. It was clear and consistent that nine groups of virulence factors are not present, and another five groups of virulence factors are equally distributed in all *S. flexneri* strains. Among the 17 virulence factors that are differentially distributed in the studied strains, chaperon usher, type 4 pili, flagella, T3SS, T6SS, siderophore mediated iron uptake, and heme-mediated iron uptake are abundantly present in the genomes of all strains. As for these seven groups of virulence factors, T3SS shows most distinct differences between sensitive strains (158 VFs) and resistant strains (207 VFs) on average. As previously reported, T3SS is a group of specialized protein export systems utilized by bacteria to effectively exploit eukaryotic hosts and contributes to bacterial adherence, invasion, and manipulation of the host's intracellular trafficking and immune systems [10]. Thus, in the multi-resistant *Shigella* strains, high number of virulence factors in T3SS groups could be very likely to occur at the same time. In fact, virulence mechanisms functioning to overcome host defence systems and antibiotic resistance are necessary for bacteria to survive antimicrobial treatments. Their collaborative work facilitates the MDR *S. flexneri* strains to adapt to and survive in competitive and demanding environments [29]. In addition, principal component analysis incorporating antibiotic resistance profile and 17 functional groups of virulence factors also showed that sensitive strain *S. flexneri* strain S15054 is isolated from other strains and most closely related to another sensitive strain S13016. All other resistant strains were all closely clustered together. Thus, from statistical point of view, it was also shown that virulence and antibiotic resistance are closely and positively correlated [12]. However, it should be notified that the spread of the two low resistant isolates in Figure 4 is a bit large. More *S. flexneri* strains should be included in future to further validate the claim.

Unique genes associated with sensitive and resistant strains were also identified based on the gene presence and absence table generated by core-/pan-genome analysis. It was found that all multi-drug resistant *S. flexneri* strains uniquely harbors four Tn7 transposon genes (*tnsA*, *tnsB*, *tnsC*, *tnsE*) and a trimethoprim resistance protein, which reflects that these strains are probably and comparatively more plastic and versatile at genome level and are more capable of acquiring resistance [30]. On the other hand, sensitive strains uniquely have type IV pill related genes (*dgcE*_1, *dgcE*_2, *dgcE*_3), genes involved in adherence and invasion (*dnaK*_2, *dnaK*_3), and the HTH-type transcriptional regulator gene *pgrR_3* that is responsible for iron uptake. All these features emphasize that sensitive strains are more likely have specialized tools to exploit cells for reproduction. Studies confirmed that bacterial strains acquiring antibiotic resistance have a lower growth rate and are less transmissible than their susceptible counterparts [31].

Except for the interplay between virulence and resistance, several studies also proposed that antibiotic resistance is linked with bacterial intracellular and environmental persistence. In specificity, antibiotic-resistant strains such as *Escherichia coli* have been reported to survive longer in macrophages [32]. Further study confirmed that resistance to antibiotics and to immune system are interconnected [33]. Moreover, Vogwill *et al.* showed that survival of antibiotic and environmental stressors is positively correlated while specific mechanisms are unrelated in *Pseudomonas* strains [34]. Thus, survival and resistance could have potential interactions in bacteria. However, it was also reported that antibiotic-resistant fecal enterococci did not survive longer than antibiotic sensitive strains [35]. The possession of the antibiotic resistance plasmids in *E. coli* did not promote bacterial survival under starvation conditions, neither [36]. Considering the controversial conclusions, it would also be interesting to investigate this relationship via theoretical analysis, which could provide more insights into this issue and support for experimental studies in future.

## 5. Conclusions

15 newly sequenced *S. flexneri* genomes isolated from clinical samples were assembled, annotated and compared by following several standardized genome analysis pipelines [16],[19],[20]. We then identified strain-specific differences in the gain and loss of putative virulence factors in this preliminary study. In addition, abundance of certain functional groups of virulence factors is positively correlated with the extent of antibiotic resistance based on the comparison of the highly resistant and susceptible strains. Several groups of virulence factors were highlighted due to their tight relationships with strong resistant phenotypes, such as chaperone usher and T3SS, *etc.* Although virulence and resistance develop on different timescales and share no much common mechanisms, they may share some common characteristics [29]. Thus, antibiotic resistance and virulence are likely to have synergistic effects toward efficiently exploiting host cells in order to reproduce and transmit extensively. However, association between virulence and resistance is an increasing problem and the answer to this question is becoming more beneficial for pathogenic bacteria [29]. This study provides a starting point to address the question of how virulence and antibiotic resistance may interplay in *Shigella flexneri* by looking into the subtle classification of virulence factors into 31 functional groups. Although the result would be much more convincing if we can incorporate other *Shigella flexneri* genomes from the public database (1121 sub-strains in PATRIC database version 3.5.39) into the study, antibiotic resistance and susceptibility phenotype data for these strains are largely missing, which greatly hinders the understanding of the interactions between the two factors. Thus, in further studies, more antibiotic resistance phenotypes should be deposited into database, together with virulence phenotypes and genomic data. In addition, fitness costs should also be incorporated to tackle the intriguing relationship among virulence, stress resistance, and antibiotic resistance from the bioinformatics point of view.
