# Supplementary material for: Comparative genome analysis of 15 clinical Shigella flexneri strains regarding virulence and antibiotic resistance
Source: AIMS Microbiol. 2019 Aug 13;5(3):205–22. doi: 10.3934/microbiol.2019.3.205 (PMC6787350; doi:10.3934/microbiol.2019.3.205)
Supplement: Supplementary file 1 [file microbiol-05-03-205-s001.pdf]

AIMS Microbiology

http://www.aimspress.com/journal/microbiology

AIMS Microbiology, 5(3): 205–222.
DOI: 10.3934/microbiol.2019.3.205
Received: 20 June 2019
Accepted: 07 August 2019
Published: 13 August 2019

*Research article*

# Comparative genome analysis of 15 clinical *Shigella flexneri* strains regarding virulence and antibiotic resistance

**Liang Wang[1,2,†], Zuobin Zhu[3,†], Huimin Qian[4], Ying Li[5], Ying Chen[5], Ping Ma[5,6] and Bing Gu[5,6,*]**

[1] Department of Bioinformatics, School of Medical Informatics and Engineering, Xuzhou Medical University, Xuzhou 221000, Jiangsu China
[2] Jiangsu Key Laboratory of New Drug Research and Clinical Pharmacy, Xuzhou Medical University, Xuzhou 221000, Jiangsu China
[3] Department of Genetics, School of Life Sciences, Xuzhou Medical University, Xuzhou, Jiangsu, China
[4] Jiangsu Provincial Center for Disease Control and Prevention, Nanjing 210009, China
[5] Medical Technology School of Xuzhou Medical University, Xuzhou 221004, China
[6] Department of Laboratory Medicine, Affiliated Hospital of Xuzhou Medical University, Xuzhou 221006, China

† These two authors contributed equally.

* **Correspondence:** Email: gb20031129@163.com.

**Table S1** Theoretical predication of antibiotic resistance genes (ARGs), virulence factor genes (VFGs), and plasmids in 15 clinical *Shigella flexneri* strains, together with multi-locus strain and plasmid typing.

| Sample Name | Species | Serotype | MDR | MLST | Plasmids | pMLSTs | Resistance Genes | Virulence Genes |
|---|---|---|---|---|---|---|---|---|
| S13016 | *Shigella flexneri* | F2a | 0 | ST-245 | NA | NA | NA | sigA, lpfA, pic, gad |
| S13028 | *Shigella flexneri* | F2b | 9 | ST-245 | ColRNAI | IncN[ST-1], IncF[F27*:A-:B-], IncI1[ST-16] | tet(A), tet(B), dfrA1, catA1, blaTEM-1C, blaOXA-1, blaCTX-M-55, aadA24, aac(3)-Iid | sigA, gad, lpfA, pic, virF, sepA, capU, ipaD |
| S13048 | *Shigella flexneri* | F1a | 6 | ST-245 | ColRNAI | NA | aadA24, strA, strB, sul2, dfrA1, tet(B), catA1, blaOXA-1 | pic, lpfA, sigA, gad |
| S13068 | *Shigella flexneri* | F2b | 7 | ST-245 | IncX1, ColRNAI, IncB/O/K/Z | IncF[F27*:A-:B-] | aadA1, strB, sul3, dfrA1, blaCTX-M-14, blaTEM-1B, tet(A) | sigA, gad, lpfA, pic, virF, sepA, capU, ipaD |
| S13073 | *Shigella flexneri* | F1a | 9 | ST-245 | ColRNAI, IncB/O/K/Z | IncF[F27*:A-:B-] | aadA24, aadA5, aac(3)-Iid, mph(A), sul1, dfrA17, dfrA1, tet(B), catA1, blaCTX-M-14, blaOXA-1 | sigA, gad, lpfA, pic, virF, sepA, capU, ipaD |
| S13091 | *Shigella flexneri* | F2a | 9 | ST-245 | ColRNAI, IncB/O/K/Z | IncF[F27*:A-:B-] | aadA24, aadA5, aac(3)-Iid, mph(A), sul1, dfrA17, dfrA1, tet(B), catA1, blaCTX-M-14. blaOXA-1 | sigA, gad, lpfA, pic, virF, sepA, capU, ipaD |
| S13109 | *Shigella flexneri* | F2a | 8 | ST-245 | ColRNAI | IncF[F27*:A-:B-] | aadA24, strB, strA, sul2, dfrA1, tet(B), catA1, blaOXA-1 | sigA, gad, lpfA, pic, sepA, capU |
| S13126 | *Shigella flexneri* | F2a | 9 | ST-245 | ColE10, ColRNAI | IncN[Unknown ST], IncF[F27*:A-:B58*] | fosA, aadA24, strA, strB, rmtB, sul2, dfrA1, tet(B), tet(A), catA1, floR, blaCTX-M-55, blaTEM-1B, blaOXA-1 | sigA, gad, lpfA, pic, virF, sepA, capU, ipaD |

*Continued on next page*

| Sample Name | Species | Serotype | MDR | MLST | Plasmids | pMLSTs | Resistance Genes | Virulence Genes |
|---|---|---|---|---|---|---|---|---|
| S14007 | *Shigella flexneri* | F2a | 6 | ST-245 | Col(BS512), ColRNAI | IncF[F103*:A-:B58*] | aadA24, strB, strA, sul2, dfrA1, tet(B), catA1, blaOXA-1, blaCTX-M-15 | sigA, gad, lpfA, pic, virF, sepA, capU, ipaD |
| S14013 | *Shigella flexneri* | F2a | 7 | ST-245 | Col(MG828), ColRNAI, IncB/O/K/Z | IncF[F27*:A-:B-] | aacA4, aadA24, dfrA1, tet(B), catA1, cmlA1, blaCTX-M-14, blaOXA-1, aac(6')Ib-cr | sigA, gad, lpfA, pic, virF, sepA, capU, ipaD |
| S14046 | *Shigella flexneri* | F1a | 7 | ST-245 | ColRNAI | IncF[F2*:A-:B-] | aadA24, strB, strA, aac(3)-IId, aadA5, fosA, mph(A), sul2, dfrA1, dfrA17, tet(B), catA1, blaTEM-1B, blaCTX-M-15, blaOXA-1 | sigA, gad, lpfA, pic, virF, sepA, capU, ipaD |
| S14131 | *Shigella flexneri* | F2a | 6 | ST-245 | Col156, ColRNAI | IncF[F27*:A-:B-] | dfrA1, aadA24, catA1, blaOXA-1, tet(B) | sigA, gad, lpfA, pic, virF, sepA, capU, ipaD |
| S15008 | *Shigella flexneri* | F1a | 7 | ST-245 | Col(MG828), ColRNAI, IncB/O/K/Z | IncF[F27*:A-:B-] | aadA24, aac(3)-IId, aadA5, mph(A), sul1, dfrA17, dfrA1, tet(B), catA1, blaCTX-M-14, blaOXA-1 | sigA, gad, lpfA, pic, virF, sepA, capU, ipaD |
| S15054 | *Shigella flexneri* | F1b | 0 | ST-245 | Col(MG828), ColRNAI | NA | sul2 | lpfA, gad |
| S15097 | *Shigella flexneri* | F2a | 7 | ST-245 | Col(MG828), Col156, ColRNAI | IncI1[Unknown ST], IncF[F27*:A-:B-] | aadA24, dfrA1, catA1, blaCTX-M-55, blaOXA-1, tet(B) | sigA, gad, lpfA, pic, virF, sepA, capU, ipaD |

AIMS Press

© 2019 the Author(s), licensee AIMS Press. This is an open access article distributed under the terms of the Creative Commons Attribution License (http://creativecommons.org/licenses/by/4.0)
